# Neurocognitive mechanisms underlying autism spectrum disorders: a literature review

**DOI:** 10.3389/fpsyt.2025.1633658

**Published:** 2025-10-03

**Authors:** Shixian Liu, Dongmei Zeng, Caiying Yi, Meijun Zhu

**Affiliations:** Department of Pediatric Neurological Rehabilitation, Ganzhou Women and Children Health Hospital, Ganzhou, Jiangxi, China

**Keywords:** autism spectrum disorder, neurocognitive mechanisms, neural circuits, synaptic function, genetic factors

## Abstract

**Introduction:**

Autism spectrum disorder (ASD) is associated with complex neurocognitive alterations that impact social cognition, executive function, and sensory processing. Understanding these mechanisms is essential for advancing diagnosis and treatment strategies.

**Methods:**

We conducted an integrative review of recent findings from structural neuroimaging, functional connectivity, molecular biology, cognitive studies, genetics, and epigenetics. Interdisciplinary approaches were summarized to examine regulatory mechanisms involved in neural development and function, as well as their impact on ASD pathophysiology.

**Results:**

Evidence from brain imaging studies demonstrates notable structural changes and disrupted neural circuit connectivity, affecting both local and global communication within the brain. Neurotransmitter system dysregulation, particularly involving glutamatergic and GABAergic pathways, contributes to excitation-inhibition imbalance, a hallmark of ASD. Genetic and epigenetic research highlights familial inheritance patterns and gene-environment interactions regulating neurodevelopment. Advancements in neuroimaging biomarkers have improved early diagnosis, while customized treatment approaches targeting specific neurobiological elements are associated with better clinical outcomes.

**Discussion:**

This review underscores the importance of a multidimensional framework that integrates imaging, molecular biology, and genetics in understanding ASD. The findings emphasize the heterogeneity of ASD and point to the necessity of individualized treatment strategies. Future research should prioritize early biomarker identification and the development of personalized therapies responsive to neural system complexity and diverse treatment outcomes.

## 1 Introduction

**Figure 1:**
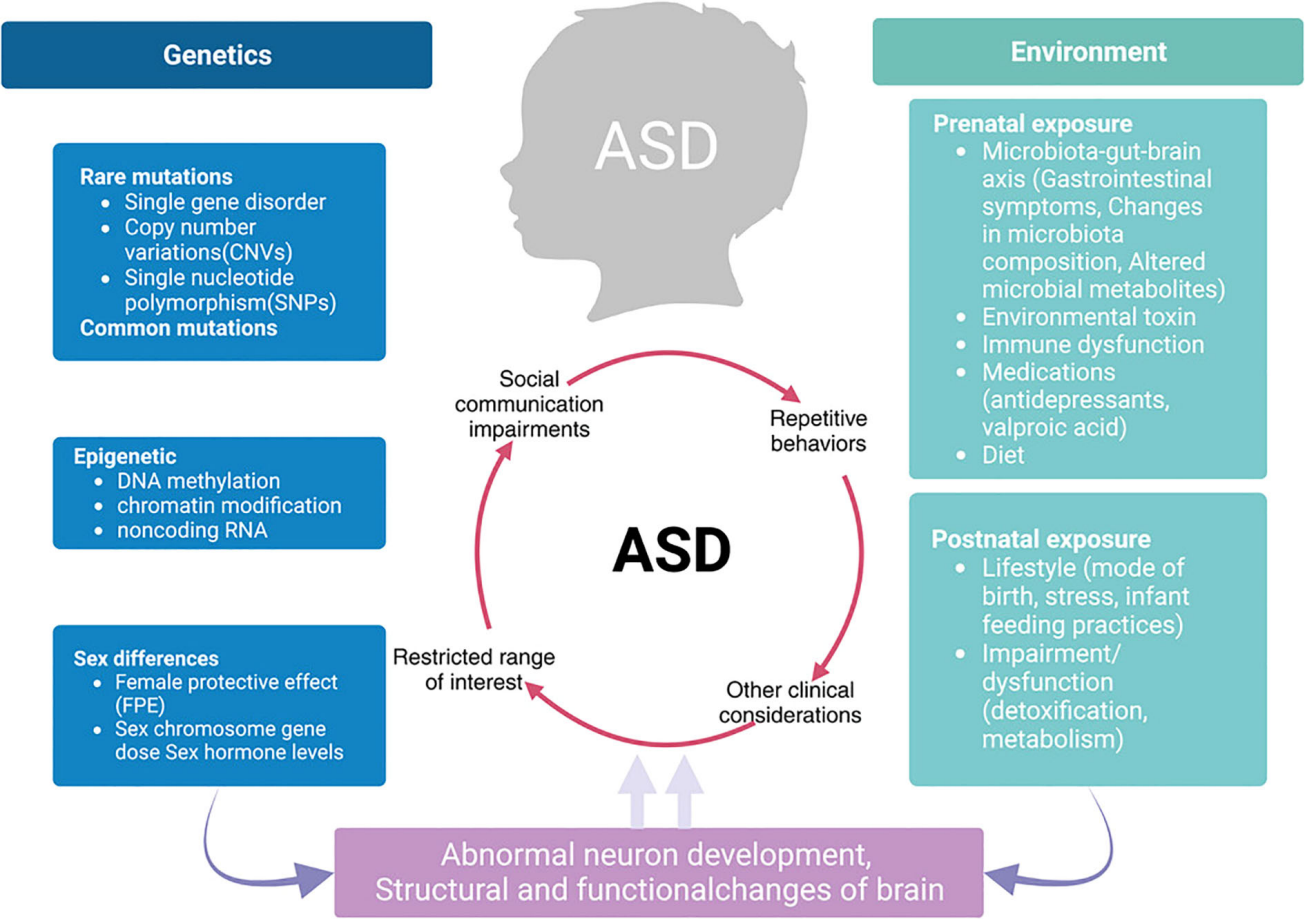
Integrated model of ASD pathophysiology linking genetics, environment, and neurodevelopmental outcomes.

Autism spectrum disorder (ASD) describes a multifaceted phenomenon with lasting deficits in social communication, interactions, and restricted and repetitive behaviours. The prevalence of ASD is said to be steadily increasing, and recent epidemiological research suggests that ASD creates enormous public health challenges (1). Current advances in neuroscience have changed our understanding of the origins of ASD by exposing the interplay of genetic, environmental, and neurobiological factors responsible for its heterogeneous manifestation (2). Based on the image presented in **Figure 1**, ASD has a complex formulation since it not only includes differences in altered neural connectivity but also, and most importantly, differences in cognition and behavioural responses which are developmental changes that occur over time.

Various techniques like neuroimaging, genetic studies, and cognitive science have attempted to analyse the neurobiological correlates of ASD. More recent structural and functional neuroimaging studies have identified certain areas of the brain exhibiting atypical organisation in individuals with ASD, mainly in the regions associated with the processing of social and sensory information (3, 4). Cortical thickening has been reported (5), altered functional connectivity patterns (6), and changes in neural circuits dynamics (7). The interplay between neuroimaging and genetics has provided insight into the biology of ASD. The genetic studies have identified some candidate risk genes and proposed molecular mechanisms for synaptic, neural circuit, and activity dependent plasticity architecture (8, 9). A contemporary avenue of research focuses on the neurobiological correlates of the particularities in cognition in ASD. Some of them uncovered specific areas of difference in the social, executive, and sensory areas of cognition (10). New explanatory approaches to the phenomena of ASD have appeared due to the conjunction of cognitive neuroscience and neurobiology. These approaches emphasise the damage of particular neural circuits associated with the behavioural and cognitive manifestations of autism (11). Furthermore, ASD has been demonstrated to be explainable in the context of social cognition (12), as well as through the prism of sensory (13) and executive (14) processes.

The wide variation in the presentation of ASD leads to severe difficulties in both research and therapy. Recently, efforts have started investigating biological subtypes of ASD which might assist with the development of tailored therapies (15). Other outlines like the gut-brain axis (16), immune system related deficits (17), and oxidative stress (18) also seem to be emerging as being crucial but require more focus. Moreover, focused research on developing biomarkers for early diagnosis and on the maintenance of therapeutic interventions is critical (19). While these mechanisms are important, they need to be meticulously exploited to enable clinicians to implement more comprehensive and effective ASD interventions and therapeutic measures. Despite all the progress that has been made, there are still many facets that are not yet comprehensively understood, making the life and complexities surrounding the disorder all the more difficult. Advances in research infrastructure, including modern neuroimaging (20), innovative genetic approaches (21), and even artificial intelligence analysis (22), have attempted to meet the task of examining the neurocognitive peculiarities of people with ASD. All of these details may translate into better options for intervening in and managing the problems surrounding the disorder.

## 2 The basis of the neurobiology

### 2.1 Structural brain abnormalities

**Figure 2:**
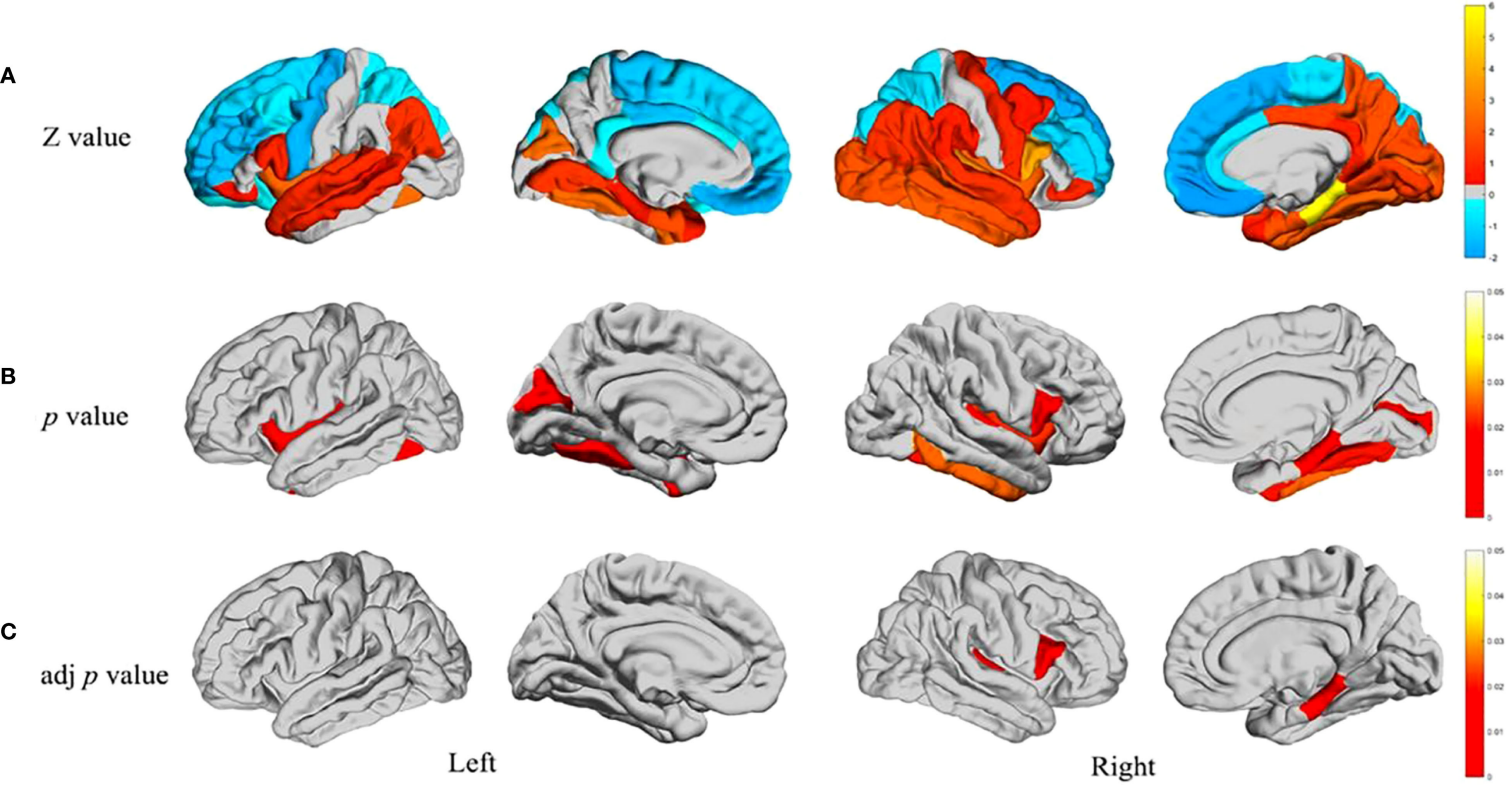
Developmental trajectory of structural brain abnormalities in ASD **(A)** Differences between individual groups regarding the structural covariance between the cerebellum and 34 other brain regions in each separate hemisphere. Warm colours in the Z-test results are reflective of having greater covariance versus the rest of the ASD sample who are shown by cool colours where covariance was low. As a result, these Z-values are reflective through the colour bar wherein Z-values calculated through Z-test are represented. **(B)** Areas of the brain characterised by significant intergroup differences in structural covariance (p < 0.05). **(C)** Intergroup differences in p values controlled for FDR (p < 0.05). Novel techniques of neuroimaging have also shown remarkable changes in the subcortical regions and the organisation of white matter. Diffusion radiomics approaches have documented specific patterns of white matter organisation in children with ASD, which help to shed light on differences in structural connectivity (23). These discoveries are crucial for the further investigation of how changes in structural connectivity may underlie the behavioural and cognitive features associated with ASD. Treatment effectiveness provided these structural MRI studies of the brain manifests some features that appear stable across different cultures and ages, but the features of alteration of the brain seen in ASD (3) are highly diverse. These neuroimaging findings have further clinical value which is corroborated by research on the MRI diagnosis of brain ASD in children (24). The cerebellum is a critical structure in the pathophysiology of ASD and has an abnormal structure and function and multitude of other studies. Specific disruptions in cerebellar ASD specific patterns which have been found van der Heijden et al. (25) suggest controversy regarding both motor and cognitive deficits. In social cognition and emotion Mapelli et al. (26) suggest that the cerebellum is involved much more than motor control. The focus of more recent work is on cognitive emotion and its implications on ASD as a whole behavioural phenotype Rudolph et al. (27). The aforementioned abnormalities at the cellular level are accompanied by structurally abnormal ASD neuronal and glial cell population. Rudolph et al. (27) notes the existence of both grey matter microstructure and white matter microstructure for ASD pathology and advances in molecular imaging clearly describe changes. Matuleviciute et al. (28) have recently highlighted changes in microglial cells; initiatives treating autism from a neurodevelopmental angle amplifying the capsule of microglial cells in regard to ASD. Understanding of biological origins that underlay the structural changes that have been observed can be achieved via these findings.

Developments in neuroimaging, especially in the use of magnetic resonance imaging (MRI), have uncovered important structural abnormalities in the brains of people suffering from ASD. These changes occur across many regions of the brain and are evident at the earliest stages of human development. Such features add to the neurobiological complexity of ASD. As shown in **Figure 2**, the structural brain abnormalities in ASD appear to be present and progress through multiple stages of development and across various neural systems, resulting in a phenotypic manifestation. One of the clinical defining features of an individual with ASD is that they have an abnormal cortical thickness. Recently, one comprehensive analysis Shen et al. focus on development demonstrated was a region-based approach for both cortical expansion and shrinkage for the process in the various stages described earlier (5). Such alterations stand out especially in regions related to social cognition, language, and executive functions. It has been shown through longitudinal neuroimaging studies that emerging differences in cortical thickness undergo at a comparatively earlier stage in children who are diagnosed with ASD than in typically developing individuals and follows a different path (87, 88). The varying rates at which these structural changes occur throughout the different stages of development indicate the existence of key time periods in development when neural circuits are susceptible to damage. A recent multimodal meta-analysis (29) combining 23 functional imaging and 52 structural MRI studies provided large-scale evidence of convergent abnormalities in ASD. The study reported decreased resting-state activity in the insula and ACC/mPFC, alongside increased gray matter volume in the middle temporal gyrus and olfactory cortices, with overlapping alterations particularly in the left insula. These findings highlight the insula and ACC/mPFC as core regions implicated in both structural and functional pathology of ASD, supporting the notion that default mode network dysfunction and atypical motor/sensory processing contribute fundamentally to ASD neurobiology.

The intricately woven macroscopic and microscopic abnormalities of ASD require targeted approaches that integrate both of these techniques. This understanding is pivotal for developing more precise diagnostic and therapeutic strategies. Further research is required to establish a correlation between a change in structure and a particular behaviour as well as cognition, along with how these alterations might lead to optimised treatment designs.

### 2.2 As for those with altered neural loop connectivity

**Figure 3:**
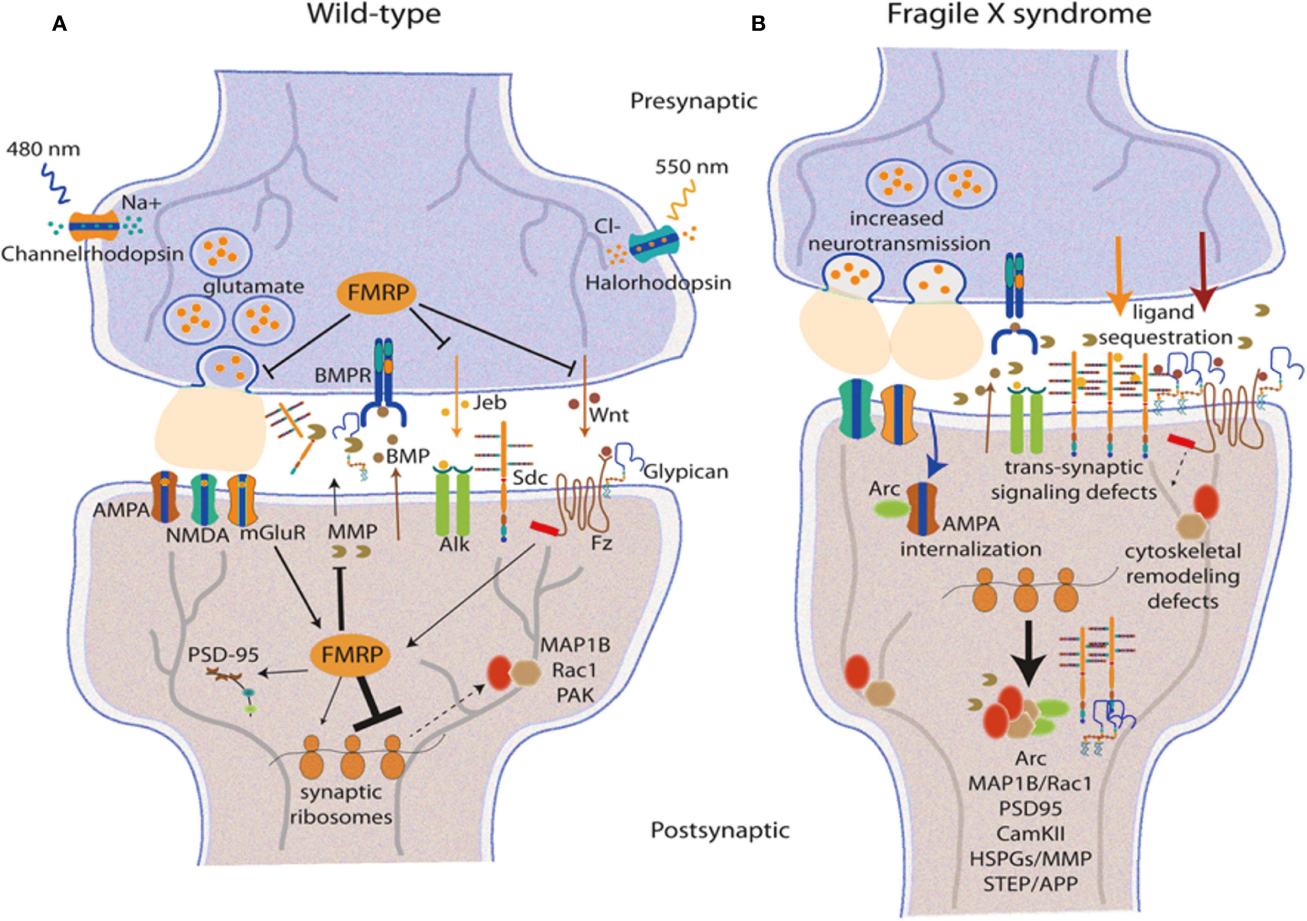
Synaptic processes and circuit organization in typical and pathological conditions. **(A)** depicts the synaptic structure of wild type, showing the presence of glutamatergic neurotransmission which includes FMRP, AMPA, and other synaptic signalling elements. **(B)** depicts synaptic pathology that is characterised by increased neurotransmission, translocated receptors, impaired receptor trafficking, and trans-synaptic signalling which is typical in many neurodevelopmental disorders. The connectivity of the prefrontal cortex is fundamental to the symptoms presented in ASD. There have been severe changes in the prefrontal functional connectivities, especially those involving the glutamatergic signalling systems and synaptic interactions (30). The more pronounced areas are the posterior cingulate cortex where, in resting state, abnormalities are constantly observed due to receptor and synaptic remapping (31). The neurogenetics of altered functional connectivity have shown subtypes of ASD to have certain distinct patterns of synaptic protein abnormalities (6). Subcortical circuit impairment of function, including that in the corticostriatal pathway, has become one of the hallmarks of ASD. Certain types of synaptic dysfunction, such as AMPA receptor trafficking and postsynaptic density (PSD) alterations, have been identified within the CTX-striatal synaptic pathways (32). These changes are associated with altered resting-state dynamics believed to be causatively linked to social deficits in ASD (33). Another study focused on brain entropy has opened the door to further understanding of how synaptic disruptions translate into neural dynamical features, calling “unrest while resting” a distinctive sign of ASD brain function (34).

Shifts in neural circuit connections are at the heart of ASD. Newer studies have highlighted intricate patterns of changes in both functional and structural connectivity across several brain networks. As shown in **Figure 3**, the molecular mechanisms of synaptic connectivity in Wild-type, or normal, development compared to pathological conditions such as neurodevelopmental disorders reveal the staggering complexity of neural circuits.

The contribution of glial cells to these changes in connectivity seems to be more significant lately due to the converging evidence from post-mortem brain as well as PET studies (35). Animal models have greatly contributed to the understanding of the neurobiological changes causing these connectivity alterations, particularly with regard to the ‘circuit’ level anomalies which are caused by dysfunction in synaptic proteins (36). More recently, this work has been extended to mouse models to examine the efficacy of removing the alterations in the circuitry inflicted by synaptic and circuit abnormalities (37). Also, those frontoparietal brain dynamics appear to have differences in dynamics within ASD specifically when it is comorbid with ADHD. There is some evidence for such alteration in the ensuing neural circuitry being an outcome of aberrant signalling of synaptic receptors and modulating synapses (38). A growing body of evidence highlights the molecular and synaptic contributions to the circuit-level dysfunction associated with ASD, thus raising the possibility of employing therapeutic measures on the synaptic as well as network components.

The detection of distinct patterns of alterations in connectivity at the molecular and circuit levels provides potential targets for therapeutic intervention as well as indicators for the need to develop bespoke therapies on the basis of neural circuitry malfunctioning on a case-by-case basis. Subsequent studies should aim at investigating pathways of abnormality of such connectivity and their correlations with clinical signs and symptoms.

### 2.3 Disregulation of neurotransmitter system

**Figure 4:**
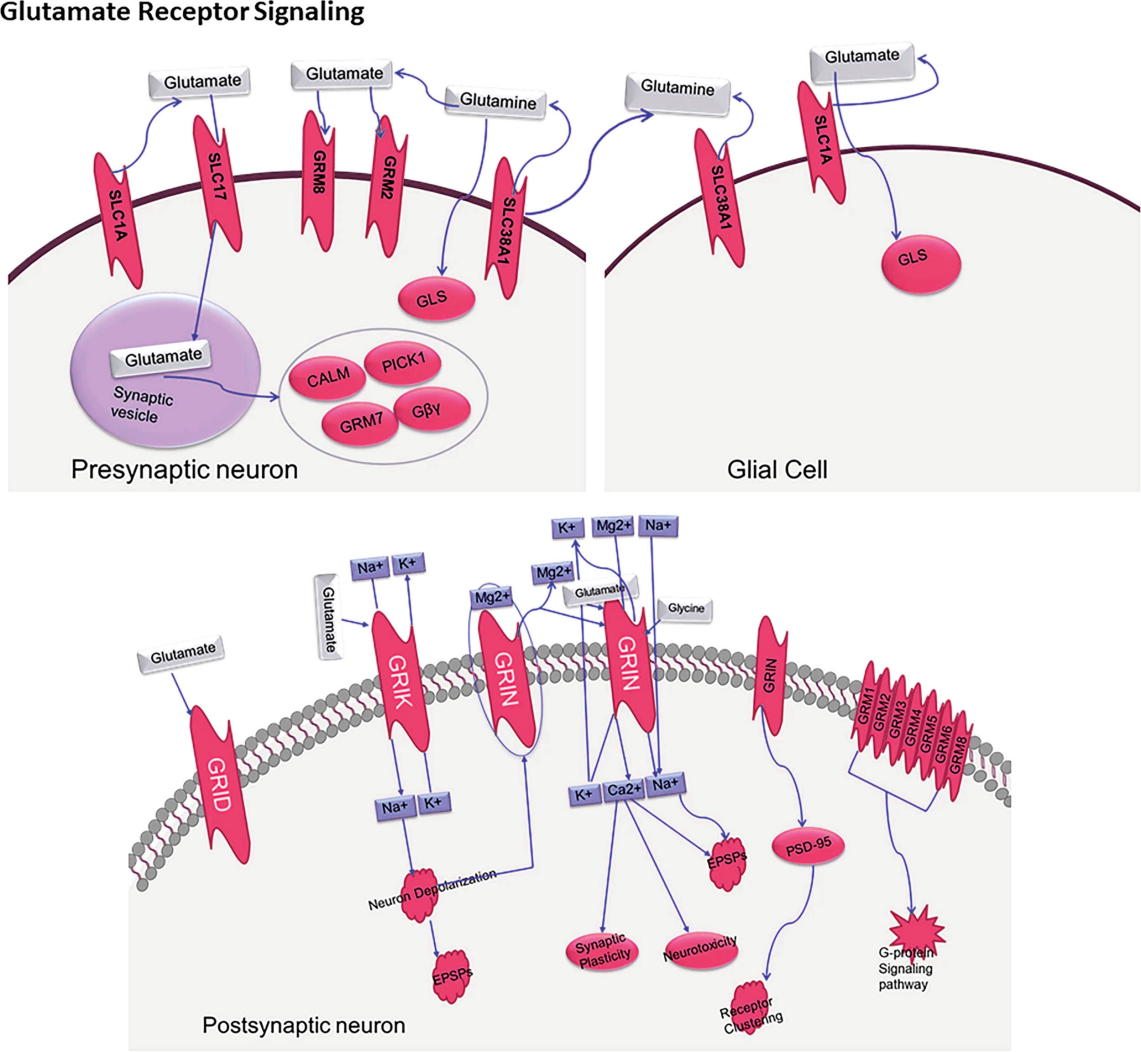
The biological processes behind glutamate neurotransmission involving key presynaptic neurons, glial cells, and postsynaptic neurons, as well as important transporters (SLC1A, SLC17A), receptors (GRIN, GRIA, GRIK), and receptors are illustrated through a molecular Glutamate Receptor sign an “SLC1A_2. SLC17A_8_Grin1_Gria1_GriK2_2_Hg Cosmc8”.

Dysregulation of neurotransmitter systems, and specifically, of glutamatergic signalling is a core component of ASD pathophysiology. As presented in **Figure 1**, neurotransmitter dysregulation occurs owing to the multifaceted relationships between presynaptic neurones, postsynaptic neurones, and glial cells. **Figure 4**, which has been integrated more recently, demonstrates the intricate glutamate receptor signalling pathway and further elucidates the presynaptic release, glial cell processing, and postsynaptic processing.

The pathophysiological mechanisms of ASD glutamate receptors’ expression and function spectrum modification have caught the attention of scientists recently. Several changes in both ionotropic as well as metabotropic glutamate are known to be increasing, changing synaptic transmission and neural circuits. Several genetic analyses have pointed out certain mutations within the genes coding for certain glutamate receptors and other associated proteins which strongly indicate ASD pathophysiology, Nisar et al, (9). Glutamate signalling and immune system functionality interplay creates a feedback loop of target engagement which facilitates the neural circuit and behavioural alterations. ASDs exhibit a notable change in the activity of neurotransmitters during the process of neuroinflammation and microglial activation, and this can be shown through the mechanisms of the neuroimmune system. Neuroglial moderation relates strongly with the rise of glutamate, neuroinflammation and synaptic activity; hence making microglia one of the leading players in neurotransmitter release and neuromodulation (32). A novel idea, pointing to the power of the immune-neurotransmitter interface with regards to efferent and afferent inter neural activities of the body, was reported by Moradi et al. (39). Most of the recent work done is around the focus of gut microbiota and their influence over the management of the neurotransmission activity in the organism. It was demonstrated that gut microbiota influence both the synthesis and catabolism of neurotransmitters which in turn affects the central and peripheral nervous systems functions (16). The microbiota-gut-brain axis is one of the most potent mechanisms through which peripheral signals modulate central neurotransmitter activities (40) with neuropeptides being the most integrated elements of this intersystem communication (41).

The influence of the melatonin system, in the context of ASD, extends beyond sleep patterns as it affects multiple neurotransmitter systems. Melatonin signalling is related to neural function and development; thus, their disruption contributes to several behavioural manifestations of ASD (42). In addition, novel relationships of these phenomena with stress responses, mast cell activation, and neurotransmitter function indicate that there is a high level of interdependence between these systems (43). We suspect that this multidimensional pattern of neurotransmitter system dysregulation clearly manifests in individuals who are diagnosed with ASD within the context of glutamate signalling, which is why systems to be used have to be integrated into a single complex action to treat autism. And these hypotheses are more likely to significantly enhance the understanding of the powerful players that trigger the underlying neurobiological systems of ASD. It will be useful for subsequent studies to identify the spatiotemporal aspects of these interactions and the rational therapeutic effects within the integrated systems.

### 2.4 Early brain overgrowth theory

One influential framework in ASD neurobiology is the Early Brain Overgrowth Theory, which posits that autism is characterized by a period of accelerated brain growth during the first 2–4 years of life, followed by atypical deceleration and disrupted neural pruning. Early MRI studies revealed enlarged total brain volume in toddlers with autism, particularly in the frontal and temporal lobes, regions associated with social cognition, language, and executive function (44–46). Longitudinal neuroimaging has confirmed these atypical trajectories, with initial cortical thickening and expansion in early childhood followed by abnormal thinning in later development (47). More recent work using predictive imaging demonstrated that early brain overgrowth patterns, especially in cortical surface area, could forecast an ASD diagnosis as early as 24 months of age (48). The latest multimodal meta-analysis (29) further supports this theory, reporting increased gray matter volume in the middle temporal gyrus and insula in children with ASD, but normalization or decline in adulthood. This developmental pattern aligns with the hypothesis of premature and excessive neural proliferation followed by atypical pruning, potentially explaining the disrupted connectivity and imbalance between excitation and inhibition observed in ASD. These findings suggest that ASD is not a static condition but rather a dynamic neurodevelopmental disorder, where early pathological brain overgrowth sets the stage for later structural and functional abnormalities across the lifespan.

## 3 Abnormal cognitive function

### 3.1 Social-cognitive impairment

One of the most prominent features of ASD is social cognition deficits, which reflect their unique neural processing of social information and their unusual behavioural responses to social interactions. Recent advances in neuroimaging have shown that there are marked changes in the social brain network, particularly in areas responsible for face and emotion processing as well as social reward (49). Such neural differences explain the prominent impairment in social interaction and communication that so many individuals with ASD struggle with.

Altered activation patterns in reward-related regions of the brain have been observed during social tasks, and this has been investigated in the context of social reward. A unique study by Baumeister et al. (12) comprehensively documented that those with ASD differ in processing social and monetary rewards, indicating that it is not the lack of ability to process rewards that is absent but there may be other reasons that could impair social reward valuation. This could contribute to reduced social motivation frequently seen in people with autism and could help understanding what therapies could be given to improve social participation. Aspect of social cognition that is impacted by ASD is the processing of emotional prosody. (50) have shown that interoceptive awareness could serve an emotional prosody recognition function, thus pointing toward therapeutic intervention methods that enhance interoceptive awareness. In addition, research on self-processing has uncovered some self-referential thoughts that are particular to ASD that can exacerbate social and perspective taking difficulties (51). The developments in social motivation related specifically to people with ASD have improved the tools used for assessments in the field. (52) appears to suggest the Social Motivation Interview as an example of a tool that has been developed to capture the more complex social interest and motivation people with ASD have. This is a useful contribution to the existing literature on the positive valence system which has identified reward and motivation abnormalities in ASD (53). The research analysing sex differences in ASD has also found these differences in the neuroanatomy of the social cognition components that may serve as sex-specific proxies for social processing differences (87; 89). These results indicate the multidimensional nature of social cognition deficits in ASD where several neural structures and various levels of cognition are involved. Identifying these mechanisms is economically important because they facilitate the development of specific measures aimed at social cognitive dysfunction in ASD patients.

### 3.2 Defective executive function

Executive function deficits represent a notable example of cognitive impairment which is commonly found in patients coping with ASD. Unfortunately, there remains a decline in cognitive flexibility, proper planning, working memory, and the ability to control actions accordingly, all of which are fundamental executive functions that impact adaptive behaviour performance (10). The most recent meta-analytic studies have documented these patterns of impairment, and they seem to vary across age groups and levels of impairment within a cognitive domain. The adolescence stage of life is especially important for executive functions because the brain regions responsible for supporting flexible cognition in individuals with ASD are distinctly engaged. Specific deficits in cognitive flexibility as well as the associated neural circuitry need to be defined and treated during this critical period of development (14). Additionally, perceptual category learning studies suggest that people with ASD uniquely process and adapt to new information (54).

The interplay of deficits in executive function and social processing within ASD has been studied extensively with regard to social cognition as well as neurocognition. This disorder was found to differ from other neurodevelopmental disorders by certain patterns of cognitive dysfunction, especially in the domain of executive control and social information processing (55). Furthermore, systematic reviews of predictive ability in ASD noted that there are changes in predictive processing which are associated with both executive function deficits and broader cognitive difficulties (56). More recent works focus on the relationship between anxiety and deficits of executive function in ASD, concentrating on specific cognitive pathways believed to heighten vulnerability to anxiety. The argument stems from the reasoning that, especially in the case of anxiety, executive function deficits are likely to mediate between anxiety and individual cognitive processing differences (57). In addition, an inquiry into the phenomena of predictive processing led to the formulation of new biobehavioural models which incorporated executive function deficits within a complex cognitive processing framework in ASD (58). These findings together comprehensively evidence the significance of the deficits in executive functions in ASD, as well as their effects on other cognitive and behavioural dimensions of the disorder. This is of great importance in considering how to design intervention programmes for children with ASD where specific higher-order functioning deficits must be targeted.

### 3.3 Sensory and perceptual processing characteristics

Abnormalities in sensory processing are part of the core features of ASD and comprise the alteration of several sensory modalities which can interfere significantly with everyday life. Previous studies have uncovered patterns of multisensory dysfunction that are complex in nature in ASD which affect both lower-level sensory processing and higher-level cognitive tasks. More recent studies have offered novel, more encompassing explanations of these deficits in multisensory processing, especially their sociocultural behavioural manifestations of ASD (11). One area that has garnered considerable attention in ASD research is auditory sensory processing. Systematic reviews have shown that patients with ASD have distinct patterns of altered auditory processing that include basic obstacles to sound perception, speech comprehension, and even more sophisticated language skills. Such findings suggest that individuals with ASD possess structural components of the neuronal machinery responsible for the complex functioning of the auditory system, but social communication and language acquisition processes are profoundly impacted (13). Diving deeper into speech processing, certain features of auditory neurophysiology that are characteristic of ASD have been documented, suggesting the presence of language pathology processing mechanisms (59). The most recent findings pertaining to studies of sensory processing on the periphery have helped understand the phenomenon of sensory alterations in ASD. It is known that peripheral differences in sensory processing go beyond the functioning of the CNS, and encompass the peripheral sensory system, which provides a broader perspective for the understanding of sensory phenomena in ASD (60). These results are important for analysing the connections between sensory processing and repetitive activities, as some studies have shown particular biopsychological correlates of restrictive and repetitive patterns of behaviour in ASD (61). One possible method for dealing with differences in sensory perception and the associated behavioural manifestation in ASD is the use of environmental enrichment. There is evidence that claims that the changing of the environment can profoundly influence these behavioural patterns through certain biological mechanisms, which raises the consideration of new therapeutic methods concerning the systems that deal with sensory perception (62). These results, which suggest the influence of environments on ASDs, capture attention as they reveal the degree of flexibility of the systems that handle their senses. The results presented here support the notion that the changes in sensory processing in an individual with autism are central phenomena, together with the other core behavioural symptoms. With these principles set, targeted interventions to address the dysfunctions of sensory processing in ASD can be designed, making sure with the utmost regard the mechanisms that pertain to intervention.

## 4 Genetic and molecular mechanisms

### 4.1 Genetic abnormalities

**Figure 5:**
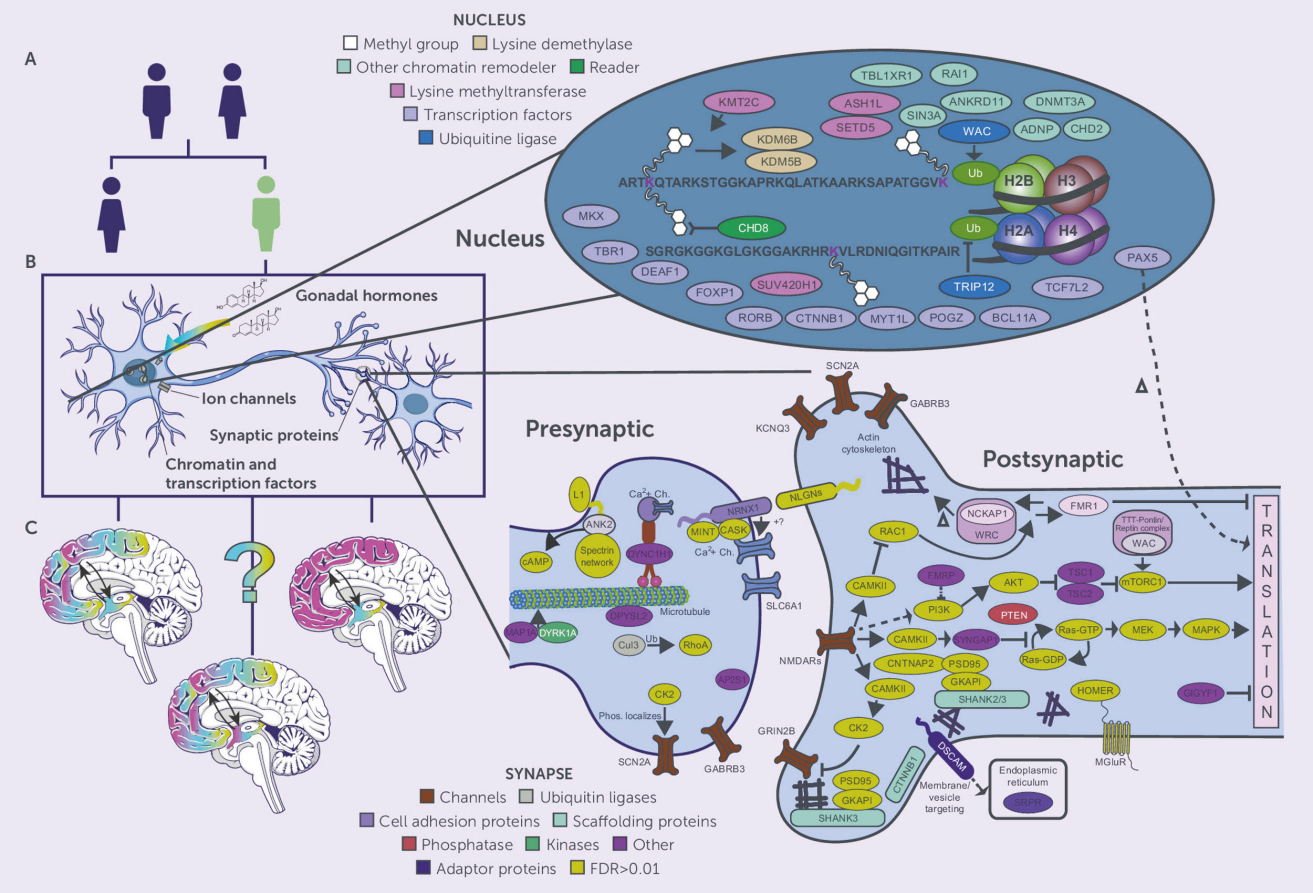
Molecular mechanisms and genetic pathways in ASD. Three parts are presented: **(A)** Sex differential genetic risk factors for ASD, **(B)** Cellular components of ASD-associated genes such as gonadal hormones, ion channels, synaptic proteins, chromatin regulators, and **(C)** Expression in specific brain regions. The detailed molecular map shows nuclear factors (chromatin remodelers, transcription factors, epigenetics) and synaptic components such as presynaptic and postsynaptic proteins, channels, and signalling cascades involved in the pathogenesis of ASD.

Genetic abnormalities play a fundamental role in the etiology of ASD, with recent advances in genomic research revealing increasingly complex patterns of genetic variation and molecular mechanisms. As shown in **Figure 5**, the molecular architecture of ASD involves multiple levels of genetic and cellular regulation, from nuclear processes to synaptic function.

Progress in the last genomic investigations has provided deeper insights into the genetic architecture of ASD. A multitude of risk genes and several molecular pathways related to neural development and synaptic activity, with a focus on chromatin modification and transcriptional regulation, have been identified (2). These findings have revealed nuclear and synaptic proteins, and their relations depicted in the extensive protein networks molecular map such as those found at pre-postsynaptic sites. Examination of molecular processes suggests a complex network of gene interactions underlying abnormal social behaviour. There are attempts to identify specific phenomena at the micro level that explain why some individuals have a given behavioural trait of ASD and how variants in the genotype influence the processes of neural circuitry formation and synaptic activity (7). Quite important are the results on dosage-sensitive genes which point to particular expression and regulatory patterns for the processes of synaptic plasticity and development (63). There have been few publications that have described Auts2 as a major regulator of some ASD behaviours and other neural activities. They have shown that the gene controls autism-like behaviour, metabolism, and stress responses at the cellular level, elucidating the many parts of the brain a single gene can affect (18). These genetic comparisons have provided evidence of mechanisms of convergence for the genetics of psychiatric disorders which encapsulate a group of neurodevelopmental disorders (64). Machine learning techniques alongside advanced genomic analyses have assisted in defining the biomarkers and genetic interactions of ASD, pointing to new therapeutic possibilities (21). Studies have shown common genetic causes for ASD and epilepsy, which suggests shared pathogenic processes at the level of synaptic components and neural circuitry (65). The **Figure 5** molecular complexity shows how genetic alterations can impact multiple cellular activities from chromatin remodelling to the nerve synapse. These molecules work is systematic and multisystemic which is challenging, but important to understand, especially to aid the development of targeted therapies for ASD. Research should be aimed at how specific genetic alterations can result in dysfunctional behaviour and abnormal brain activity, in light of the sex-specific and region-specific expression and regulatory activities of the genes depicted in the figure.

### 4.2 Epigenetic regulation

**Figure 6:**
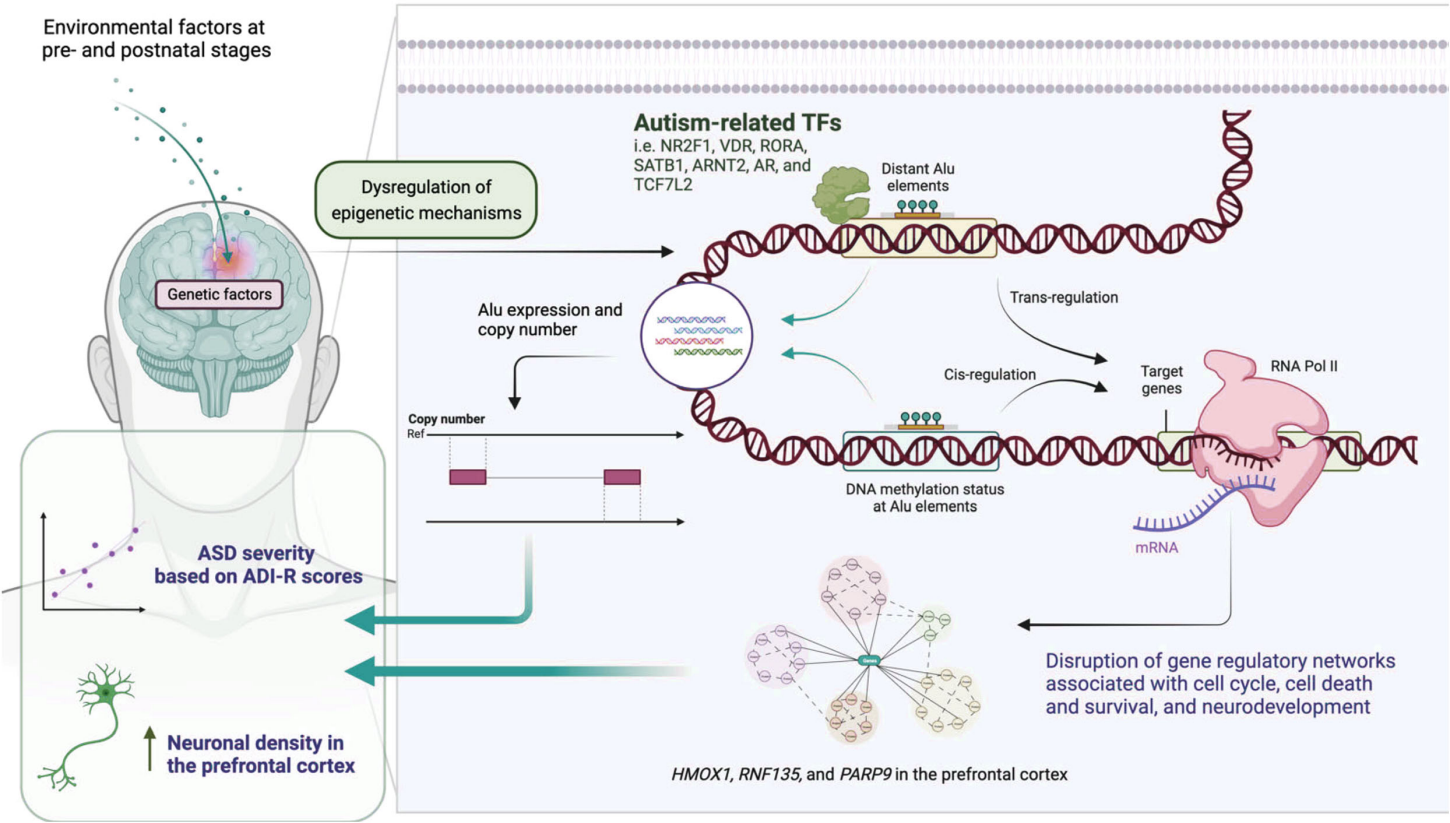
Epigenetic regulatory mechanisms in ASD.

Epigenetic regulation represents a crucial mechanism in the pathogenesis of ASD, involving complex interactions between genetic and environmental factors that influence gene expression without altering DNA sequences. As illustrated in **Figure 6**, epigenetic mechanisms in ASD encompass multiple levels of chromatin regulation and transcriptional control.

The most recent studies looking into the genetic components of structural and functional changes in the brain processes of an individual diagnosed with ASD emphasise the role of epigenetic changes. Research studies have shown that the atypical phenotypes expression of children with ASD may be caused by some alteration in the chromatin structure and methylation of DNA (8). These modifications to the epigenome modify the expression of neurodevelopmental processes like synaptogenesis, neural circuitry assembly, and activity-dependent changes in plasticity. The association, that is, the overlap between cognitive phenomena and genetic phenomena with features of autism has further confirmed the ascertained deficit for other epigenetic mechanisms which modulate gene expression. More recent ASD cognitive phenotype studies have shown that these phenotypes are in some way epigenetically altered where the condition is termed “gene silencing” (66). This raises other questions on the degree to which epigenetic alterations act as covariant determinants to the many knowledge and information in other cognitive domains of children with ASD. Induced pluripotent stem cells (iPSCs) can be used as advanced cellular models to study the mechanisms of epigenetics in ASD. They have enabled researchers to investigate the functioning and development of neural tissues in ASD and to find new treatment options (67). The use of iPSC technology can create very specific types of epigenetic alterations concerning their undesirable or abnormal development and activity within the neural circuits during the period of adolescence.

Investigations into certain prime proteins such as ankyrin-G have provided insights into the processes of epigenetic modifications concerning synapse functionality and neural circuit development in ASD (90). It is believed that their ‘behavioural’ neural functions can be mediated due to the ‘epigenetic’ changes that have been carried out on them. Epigenetic changes and the proteins that form the synapse provide a means through which ASD can be treated efficaciously. Different forms of epigenetic changes that are modulating the pathway of ASD have signs indicating sex- and gender-based differences. Studies are identifying where specific cognitive deficits may serve as a potential moderator in sex/gender differences of ASD point to more intricate systems of epigenetic changes and cognition. These findings concerning the phenomenon of sex in combination with the phenomena of epigenetics particularly draw attention to the need to develop treatment strategies for ASD. Developing a deep understanding of these intricate epigenetic processes is essential to accurately construct interventions for effective treatment of ASD. The relationship between chromatin structure, the control of gene expression, and the activity of the synapse enables us to envision multiple ways in treating such conditions. Further studies should be aimed towards how certain epigenetic changes cause irreversible changes leading to the development of ASD and how these changes can be reversed or altered pharmacologically.

### 4.3 Gene-environment interactions

The gene-environment interaction is one of the most important aspects of ASD as it epitomises the interaction between one’s genetics and their surroundings. Studies done on maternal autoantibodies have shed a great deal of light on the interaction between a mother’s immune factors and fetal neurodevelopment which suggests certain pathways where maternal antibodies can alter brain structure and heighten the chances of ASD (68). Findings such as these are crucial to grasp because they define the significance of the environment before birth in the expression of ASD. It integrated the fact that the neurodevelopmental impacts of stress during early childhood, especially for children with a genetic risk of ASD, are profoundly high (69). It is maintained that hormones from distress and genetic material interact as an explanation for differences in brain structure and functions which ultimately develop differing symptoms of autism. Stress adds genes, which come first, certainly interacting between stress hormones and brain material. It also clarifies the structure makeup in the brain which is able to evolve different functions that control various symptoms of autism.

The interplay between immune system dysregulation, oxidative stress, and the environment constitutes important factors influencing the pathogenesis of ASD. In some individuals with certain genetic variants associated with ASD, oxidative stress can interact with other genetic risks to impair neural development and functioning (17). The existence of different inflammatory subtypes within ASD adds another dimension, suggesting that the immune system and genetic risk factors are more intricately connected than previously thought (70). The sleep issues of individuals with ASD illustrate the complexity of genetic predispositions and how they both interact with and are influenced by surrounding factors. More recent work has focused on the bidirectional nature of the relationship between sleeping and ASD where sleep problems either serve as a precursor or a consequence of the disorder (71). Neurobiological work on the integration of genetics into environmental contexts for the development and functioning of the brain in the framework of the development of an ASD uses the model of the framework of twice exceptionality phenomena depicts this quite well (72). In combination, these studies beginning to approach the boundary of gene-environment interactions in ASD offer a novel framework from an etiological and heterogeneity point of view. Understanding these interactions is essential for effective intervention strategies since they can be designed in consideration of genetic and environmental risk factors of ASD.

## 5 Clinical diagnosis and treatment strategy

### 5.1 Early diagnosis

The identification of new biomarkers and the development of new diagnostic tools have made it possible to detect ASD at earlier ages than previously possible. The diagnostic approach is being enhanced with new biological markers, particularly neurophysiological ones. New magnetoencephalography (MEG) technology has emerged that may aid in the search for ASD biological markers by measuring neural circuit abnormalities and other potential diagnostic markers (73). This advancement is further supported by the attainment of EEG biomarkers which, through the investigation of neural oscillations and connectivity patterns, provide strong evidence in support of the diagnosis of ASD (74). The classification of techniques including MEG-based markers strengthening the evidence for early and accurate diagnosis of ASD has increased greatly (73).

The use of artificial intelligence and machine learning technologies has undoubtedly resulted in transformations in the strategies employed while diagnosing ASD. It has been revealed that the features of deep learning methods significantly improve the accuracy and detection rates of ASD’s intricate neurological attributes (20). One of the more unique methods is screening for ASD and evaluating the level of symptoms using retinal photographs. This method is unique in its innovation due to its effective non-invasive nature and ease of use (75). Along with these advances in technology, sophisticated approaches of text mining constructs derived from brain entropy are opening new possibilities in diagnosing ASD using neural complexity and dynamics (22). The combination of different techniques has significantly increased the ability to diagnose individuals with ASD at an early stage of its manifestation. The efforts put in towards combining sophisticated information technology with biomarkers have resulted in more sophisticated systems. The merging of neurophysiological markers, imaging, alongside the artificial intelligence-based performance expands the comprehension of the spectrum of manifestations of ASD which provides for better early detection. Supporting more than one method for early detection of specific subtypes of ASD not only enhances accuracy but permits a differential approach for timely precise and appropriate intervention and treatment.

### 5.2 Intervention method

The latest advances in treating neurodevelopmental disorders like ASD signify progress in medicine. There has been an increased understanding of the molecular and neural mechanisms of ASD, so there is now greater emphasis on developing therapeutic approaches that target an individual’s biology and genetics (76). One such novel therapeutic approach is cannabinoid-based therapies that address the neuroinflammatory components of ASD. Some evidence indicates that cannabinoids can help not only with neural signalling but also with inflammation, which could be beneficial in more advanced therapeutic interventions (77). Even today, behavioural therapies remain among the most frequently used interventions for ASD treatment, particularly for symptom management. Recently developed centred approaches that focus on understanding ASD and modifying the symptoms, particularly the restricted and repetitive behaviours, appear to be effective (78). Additionally, some learners with non-verbal ASD neurodevelopmental disorders have been reported to respond to new techniques of cognitive training, such as symbol relations training, which has shown significant improvement in the neurodevelopmental cognitive skills of the children (79). These interventions are of great importance to pre-verbal autistic children who require considerable assistance in communication and social skill development (80).

Phytochemicals extracted from dietary sources have been attracting attention as potential treatments for ASD. Plant neurotherapeutics targeting specific pathways involved in the pathophysiology of ASD have already been discovered and reported (81). Such compounds, combined with other natural therapies, adopt a more holistic approach to treatment that utilises more than one aspect of the ASD syndrome. The different methods of treatment such as precision medicine, behavioural modification therapy, and other unconventional remedies reveal the level of flexibility in seeking solutions to the problems posed by the wide spectrum of the disorder and the requirements of the person affected by autism.

### 5.3 Individualized treatment

Recent neurobiological studies have indicated that it is possible to define several separate neurological subtypes for ASD, which makes it possible to develop more targeted therapies for patients. Recently conducted research on subtypes of ASD suggests that an individual should be supplied with differentiated intervention strategies constructed on unique features of their brain’s structure and its functions (15). This information has been augmented by studies of some rationally dominating subgroups of ASD, representing the therapy’s strengths-based approach (82). Systematic reviews done on ASD have clearly pointed out that formulating treatment strategies requires consideration of both the difficulties and the gifts of the individual (83). The previously mentioned novel research concerning astrocyte neurite extensions in ASD has revealed aspects pertaining to the cellular bases of personalised treatment. Other recent works have indicated that there is a sophisticated crosstalk between these two cell types, which means that interventions would need to target particular cellular mechanisms in a person’s pathophysiology (84). This changes the emphasis to a within-person level of intervention because, with these forms of understanding, there is no way to fashion one model for all persons with the so-called neurodiversity diagnosis (85). A combination of biological understandings together with individual respects forms the foundation of a new step in approaches to personalised treatment. Such experimental models have been important in understanding how to formulate and improve treatment approaches for ASD. Neurobiological mechanisms and potential therapeutic targets can be investigated in greater detail using zebrafish models, which allow for the development of more individualised intervention strategies (86). These systems allow fast intervention testing for many approaches so that the key mechanisms targeted in specific cases are easily diagnosed. Due to much advancement in biological understanding, respect for neurodiversity, and experimental model systems, a comprehensive framework for developing truly personalised treatment approaches in ASD has emerged that respects the biological complexity of the condition and the individual variability in treatment response.

### 5.4 Limitations of current research methodologies and future direction

Despite significant progress, several methodological limitations constrain our current understanding of ASD neurobiology. First, there is considerable heterogeneity across study samples, including differences in age groups, symptom severity, comorbidities, and cultural backgrounds, which complicates cross-study comparisons. Second, neuroimaging studies vary widely in acquisition protocols and analytic pipelines, reducing reproducibility and complicating meta-analytic integration. Third, many studies rely on relatively small sample sizes, which limits statistical power and generalizability. Finally, the majority of findings remain correlational rather than causal, underscoring the need for longitudinal and multimodal approaches to establish developmental trajectories and mechanistic pathways. Future research should prioritize longitudinal and multimodal approaches that integrate neuroimaging, genetics, epigenetics, and environmental data to delineate developmental trajectories and biological subtypes of ASD. Mechanistic studies using animal models and induced pluripotent stem cells will be crucial to uncover causal pathways of synaptic dysfunction, immune–brain interactions, and gut–brain axis abnormalities. At the clinical level, artificial intelligence–driven diagnostic tools and biomarker-based stratification should be combined with intervention trials that account for neurobiological heterogeneity. Large-scale, cross-cultural consortium studies are also needed to enhance reproducibility and generalizability. Collectively, these strategies will enable more precise early diagnosis, targeted interventions, and ultimately improved outcomes for individuals with ASD.

## 6 Conclusion

Beyond advancing scientific understanding, the neurocognitive mechanisms reviewed here have important translational implications. Identifying early biomarkers of atypical brain growth and connectivity can improve early diagnosis, allowing interventions to be implemented during critical developmental windows. Insights into neurotransmitter imbalance and synaptic dysfunction inform the development of targeted pharmacological strategies aimed at restoring excitation–inhibition balance. Recognition of cognitive subdomains such as social, executive, and sensory processing deficits provides guidance for personalized behavioral interventions. Moreover, acknowledging the heterogeneity of ASD and the presence of distinct neurobiological subtypes highlights the necessity of individualized treatment strategies that align with each patient’s neural and cognitive profile. Ultimately, this knowledge contributes to improving the quality of life, adaptive skills, and long-term outcomes for individuals with ASD and their families.
